# Tackling disease‐related malnutrition in resource‐limited settings: An international position paper based on expert consensus

**DOI:** 10.1002/ncp.11310

**Published:** 2025-05-30

**Authors:** Diana Cardenas, Ines Ribeiro Ferreira, Maria Isabel Toulson Davisson Correia, Mario Barbagallo, Simon Lal, Rocco Barazzoni, Filomena Gomes, the Working Group

**Affiliations:** ^1^ Nutrition Unit Gustave Roussy Villejuif France; ^2^ NOVA Medical School Universidade NOVA de Lisboa Lisbon Portugal; ^3^ Department of Surgery Universidade Federal de Minas Gerais Belo Horizonte Brazil; ^4^ Nutrition Therapy Team Rede Mater Dei and Hospital Semper Belo Horizonte Brazil; ^5^ Médecins Sans Frontières Brussels Belgium; ^6^ Northern Care Alliance Salford United Kingdom; ^7^ Internal Medicine University of Trieste Trieste Italy; ^8^ The Micronutrient Forum Washington, DC USA

**Keywords:** malnutrition, nutrition care, resource‐limited settings

## Abstract

**Background:**

Considering the challenges of providing nutrition care in resource‐limited settings (RLSs), the International Working Group for Patients' Right to Nutrition Care (WG) organized an expert meeting to propose recommendations and strategies to promote access to nutrition care and address disease‐related malnutrition (DRM).

**Methods:**

An online survey was developed to assess barriers to providing nutrition care in RLSs and was completed by 58 respondents from low‐ and middle‐income countries between July and August 2024. During the European Society for Clinical Nutrition and Metabolism (ESPEN) Congress in Milan on September 9, 2024, a panel of 30 experts discussed the results of the survey and built consensus statements aimed at defining strategies and recommendations required to address barriers to accessing disease‐related nutrition care in RLSs.

**Results:**

The survey and expert consensus panel opinions indicated that there are barriers to delivering quality nutrition care in these settings including low or a lack of medical awareness, patient and family knowledge about DRM and its impact, nutrition risk screening and care implementation, reimbursement, medical devices, adapted diets, nutrition protocols, and access to home medical and nutrition therapy. Gaps identified included (1) epidemiological data and evidence for best practices; (2) education, training, and capacity building; and (3) strengthening health systems.

**Conclusion:**

Tackling DRM in RLSs is challenging because of the high burden of DRM and the fact that current guidelines from high‐income countries may not be fully applicable. The WG recommend a three‐step strategy to promote access to nutrition care.

## INTRODUCTION

Healthcare inequalities are more frequent in resource‐limited settings (RLSs).^1^ Given that disease‐related malnutrition (DRM)^2^ has been neglected from a policy perspective both nationally and internationally, healthcare inequality in nutrition care is even more pronounced. The United Nations Sustainable Development Goals, especially Sustainable Development Goals 2, which aims by 2030 to end all forms of malnutrition, does not discuss DRM, particularly in the hospital context. Health policies addressing the problem of DRM in community or hospital contexts are scarce.^3^, ^4^

The impact of DRM on clinical outcomes has been studied in large epidemiological and randomized controlled trials, leading to strong consensus among the scientific and medical community about its relevance and the need to implement evidence‐based, standardized nutrition care for patients at risk of malnutrition.^5^, ^6^, ^7^, ^8^ DRM increases healthcare costs because more care is needed and hospital stays are prolonged. It associates with comorbidities, delayed rehabilitation, and the risk of death. Individualized nutrition care for hospitalized medical and surgical inpatients at nutrition risk improves clinical outcomes, including survival.^6^, ^9^

The impact of DRM on outcomes and access to nutrition care in RLS has not been addressed, as the priority of nutrition interventions in these countries tends to be focused on maternal, infant, and early childhood health (https://globalnutritionreport.org/blog/tackling-malnutrition-in-a-changing-world-a-call-for-accountability-and-action/). Data on the prevalence or indicators of the extent of DRM in RLS, particularly in low‐income countries, are scarce.^5^ In Latin America, results of the nutritionDay survey provided some insight into nutrition care issues in RLS and demonstrated that the prevalence of nutrition risk in hospitalized patients is likely higher than in European countries (40% vs 30%).^5^, ^10^ However, these results included data from upper‐ and middle‐income countries (Colombia and Brazil).^11^, ^12^ This difference could be explained by the assumption that other social determinants (ie, food insecurity, lack of access and affordability of nutritious foods and medical nutrition therapy, and general socioeconomic conditions) contribute to the higher prevalence of nutrition risk in these countries. The extent and cause of DRM remain to be fully understood. In Africa, according to a scoping review, malnutrition risk (23%–74%) and malnutrition (8%–85%) prevalence are alarmingly high in adult hospitalized patients.^13^ The authors state that realities in the African context include “limited nutritional screening and assessment, poor referral practices, a unique disease burden, and probably a limited awareness of the importance and potential benefit of addressing hospital malnutrition.”^11^

One could argue that part of the way forward in tackling DRM is to address the social determinants of health and scaling up the availability of nutrition care access (ie, screening, diagnosis, assessment, medical nutrition therapy, and monitoring). The initial approach to addressing DRM in RLSs clearly requires an understanding of the barriers to nutrition care access, particularly for screening and treatment (ie, medical nutrition therapy), followed by providing recommendations and strategies to overcome these barriers. Thus, the International Working Group for Patients' Right to Nutrition Care (WG) created in 2020 and composed of experts in clinical nutrition and representatives of the main international societies organized an expert panel meeting to propose recommendations and strategies to tackle DRM in RLSs. The meeting was held during the European Society for Clinical Nutrition and Metabolism (ESPEN) congress in Milan, Italy, on September 9, 2024. The purpose of this paper is to propose recommendations and strategies to promote access to nutrition care and address DRM in RLSs according to a panel of experts in clinical nutrition.

## THE WORKING GROUP

The WG was launched to explore how the human rights–based approach can contribute to the challenges faced by the practice of clinical nutrition in increasingly demanding healthcare systems.^14^ The WG is composed of experts in clinical nutrition and representatives of ESPEN, the American Society for Parenteral and Enteral Nutrition (ASPEN), the Latin American Federation of Nutritional Therapy, Clinical Nutrition, and Metabolism (FELANPE), and the Parenteral and Enteral Nutrition Society of Asia (PENSA).

For the purpose of writing this position paper, an extended WG was created to participate in the panel meeting, discuss the results of a survey, and build consensus statements aimed at defining strategies and recommendations to address barriers to accessing nutrition care in RLSs. Experts from the nongovernmental organization (NGO) Doctors Without Borders (Médecins Sans Frontières Belgique) (https://www.msf-azg.be/fr) were invited to participate in the design of the on line survey and the expert meeting.

In this paper, the term “RLS” in clinical nutrition defines a setting where the capability to provide care for malnourished patients is limited to basic critical care resources, including nutrition therapy and trained staff. A “humanitarian organization” is an organization that has a bona fide mission to address the public health needs of underserved populations on a not‐for‐profit basis.

## THE SURVEY

Barriers to the provision of nutrition care in RLS were surveyed through an online questionnaire in five languages: Spanish, English, French, Portuguese, and Arabic. The questionnaire was designed using Google Forms and disseminated via email in collaboration with the NGO Doctors of the World. The WG methodically selected respondents according to the following criteria: healthcare professionals, humanitarian professionals, and policy makers; those working in the community, hospitals, nursing homes, or other healthcare settings and humanitarian situations; and those directly involved with DRM in RLSs in low‐ and middle‐income countries (LMICs).

The questionnaire was developed using a Levesque et al framework incorporating the dimensions of approachability, acceptability, availability/accommodation, affordability, and appropriateness.^15^, ^16^ The questionnaire consisted of 10 Likert‐type questions and was emailed in July 2024 to targeted identified respondents. Data were collected in August 2024.

The survey was designed to serve as the basis for discussion during the expert panel meeting and should not be considered as an epidemiological or research survey. The survey was the basis for the panel's deliberations during the expert meeting.

We received 58 responses from clinicians who deal with nutrition care in RLSs in LMICs (Afghanistan, Colombia, Democratic Republic of the Congo, Ethiopia, Dominican Republic, Guatemala, Guinea, India, Kenya, Paraguay, Sri Lanka, and Uruguay), 18 of whom worked in the humanitarian context. Table 1 shows the diseases related to malnutrition.

**Table 1:** Diseases according to respondent cause or association with malnutrition.

Diseases related to malnutrition	Responses
Cancer	42
Noncommunicable diseases	41
Other gastrointestinal diseases	41
Intestinal insufficiency (ie, patients requiring parenteral nutrition)	37
Intensive care patients	33
Other infectious diseases	32
Tuberculosis	32
HIV	29
Obesity	27
Postsurgical patients	3
Marasmic kwashiorkor	2
Children with developmental and growth restrictions	1
Critically ill patient	1
Failure to breastfeed	1
Hepato‐pancreatobiliary diseases and liver transplantation	1
Kwashiorkor	1
Marasmus	1
Renal diseases	1
Severe acute malnutrition	1

The higher‐consensus responses for each category are shown in Table 2 for the humanitarian and nonhumanitarian contexts. In humanitarian settings, the barrier with the greatest level of consensus was “lack of patient and family knowledge about malnutrition and its impact (98%).” In nonhumanitarian contexts, the barriers with the greatest level of consensus were “the governments do not pay for home medical nutrition therapy (85%)” and “patients/families cannot afford medical nutrition therapy (82%).”

**Table 2:** The higher‐consensus responses for each category for the humanitarian and nonhumanitarian contexts.

	Approachability	Acceptability	Availability	Affordability	Appropriateness
Humanitarian context	Lack of patient and family knowledge about malnutrition and its impact (98%) Lack of nutrition risk screening and assessment process in the institutions (eg, hospital, home care, or outpatient clinics) (76%) Poor medical awareness (mainly doctors) (65%)	Patients/families/careers understanding of medical clinical nutrition therapy (eg, tube placement or venous central catheter) (65%) Doctors’ disbelief in medical nutrition therapy (47%) Patients/families/carers’ fears of medical nutrition therapy (41%)	Lack of access to home medical nutrition therapy (88%) Lack of humanitarian aids/actors investing in this topic (82%) Lack of medical nutrition dispositive (eg, tubes/catheters) (82%) No compounding pharmacies to prepare customized enteral and parenteral nutrition (76%)	The government does not pay for home medical nutrition therapy (88%) Patients/families cannot afford medical nutrition therapy (88%) Institutions are not paid either by the government or insurance companies for medical nutrition therapy (82%) Insurance companies do not cover home medical nutrition therapy (70%)	The amount of time allocated to medical nutrition therapy by staff (88%) Time dedicated by doctors explaining the importance of medical nutrition therapy (82%) Lack of a nutrition specialist responsible for medical nutrition therapy in the institution (76%)
Nonhumanitarian context	Lack of patient and family knowledge about malnutrition and its impact (73%) Lack of nutrition risk screening and assessment process in the institutions (eg, hospital, home care, or outpatient clinics) (73%) Poor medical awareness (mainly doctors) (66%)	Patients/families’ understanding of medical clinical nutrition therapy (eg, tube placement or venous central catheter) (70%) Doctors’ disbelief in medical nutrition therapy (56%) Patients/families’ fears of medical nutrition therapy (44%)	Lack of access to home medical nutrition therapy (75%) Lack of humanitarian aids/actors investing in this topic (65%) No compounding pharmacies to prepare customized enteral and parenteral nutrition (63%) No compounding pharmacies to prepare customized enteral and parenteral nutrition (63%) Lack of a nutrition protocol established in the institutions (6%)	The government does not pay for home medical nutrition therapy (85%) Patients/families cannot afford medical nutrition therapy (82%) Insurance companies do not cover home medical nutrition therapy (75%) Institutions are not paid either by the government or insurance companies for medical nutrition therapy (66%)	The salaries paid to those carrying out medical nutrition therapy (73%) Patients' and families’ perception of the quality of service regarding medical nutrition therapy (66%) Lack of a nutrition specialist responsible for medical nutrition therapy in the institution (59%)

## THE EXPERT MEETING

The WG organized an expert panel meeting during the ESPEN congress in Milan, Italy, on September 9, 2024. The panel of experts included the members of the WG and invited clinical nutrition experts with relevant background and interest in the field. A total of 33 experts including doctors, nurses, scientists, dietitians, and a pharmacist participated in a 2‐h discussion to address specific nutrition‐related issues in RLSs and analyzed the results of the survey. The WG coordinator presented each of the survey questions. Based on the data and discussion of the WG meeting, the arguments were summarized in 10 statements on strategies and recommendations to address the identified barriers to access to nutrition care in RLSs. The experts were asked to vote by secret ballot.

### Statements

1. Even though economic barriers exist, malnourished patients should aspire to receive the best nutrition care possible (Consensus: 100% agreement).
2. Ethically, healthcare professionals, professional societies, NGOs, and international organizations from non‐RLSs have a duty to promote nutrition care for patients in RLSs (Consensus: 100% agreement).
3. Clinical nutrition societies, NGOs, and international organizations must recognize that the delivery of safe, equitable and high‐quality nutrition care in RLSs is a priority for international public health security (Consensus: 100% agreement).
4. Clinical nutrition societies should provide considerations to what should be offered (potential solutions) when resources are constrained (Consensus: 100% agreement).
5. To improve nutrition care in RLSs, the following should be implemented (Consensus: 100% agreement):
  a) Nutrition care plan (Consensus: 96% agreement).
  b) Clinical guidelines (Consensus: 100% agreement).
  c) Education and training for healthcare professionals (Consensus: 100% agreement).
  d) Research in DRM capacity (Consensus: 100% agreement).
  e) Healthcare system strengthening (Consensus: 83% agreement).

6. Clinical nutrition practice in RLSs should rely on literature from developed countries (Consensus: 74% agreement).
7. Tailoring guidelines in clinical nutrition for RLSs according to the local healthcare needs and the resources available is necessary (Consensus: 100% agreement).
8. Together, non‐RLS and RLS experts must develop adapted recommendations for the management of DRM (Consensus: 100% agreement).
9. Until sufficient research from RLSs drives locally generated clinical guidelines, adaptation of existing guidelines is essential to bring safe, feasible, and effective practices to the bedside (Consensus: 100% agreement).
10. Until sufficient research from RLSs drives locally generated clinical guidelines, new tailored guidelines are essential to bring safe, feasible, and effective practices to the bedside (Consensus: 100% agreement).

The survey analysis and discussion identified gaps in three fields: (1) epidemiological data and evidence for best practices; (2) education, training, and capacity building; and (3) strengthening health systems.

### Epidemiological data and evidence for best practices

Considering that the outcomes of nutrition intervention generated by local clinical trials are rarely available, health professionals in RLSs and LMICs rely on literature from developed/high‐income countries. However, nutrition interventions that improve outcomes in patients from resource‐rich settings may not always be necessary or available in RLSs. As an example, oral nutrition supplements or commercial enteral formulas may not be available or affordable, but well‐planned alternatives made of locally available foods, prepared with appropriate food safety conditions, may be a good alternative. Guidelines developed in resource‐rich countries can be adapted to take into account the specific local characteristics of the RLS before implementation. Moreover, clinical trials conducted within the parameters of a high standard of care may produce results that cannot be implemented or sustained locally when the health system is resource constrained. According to Griswold et al, between 2005 and 2013 only 4.7% of patients included in clinical trials were recruited from LMICs and only 0.8% from low‐income countries.^17^

Guidelines tailored to RLSs have been developed within the last 10 years in other fields, such as oncology and intensive care.^18^, ^19^, ^20^ According to Diaz et al, in the critical care setting, “adaptation of existing guidelines is essential to bring safe, feasible, and effective practices to the bedside.”^21^ Although there is no consensus standardized methodology for generating resource‐stratified guidelines (RSGs), the Breast Health Global Initiative^22^ and the National Comprehensive Cancer Network framework^17^ have demonstrated notable success in developing guidelines for RLS stratified by four resource levels: “basic, limited, enhanced, and maximal.” RSGs identify a hierarchy of interventions based on the level of economic development and offer a transparent and plausible approach to guiding treatment decisions in different resource settings.

### Education, training, and capacity building

Educational and training strategies to build disease‐related nutrition care capacity in RLSs are important because of the lack of nutrition topics in medical school curricula,^23^ the lack of postgraduate training, and limited access to continuing medical education.^24^, ^25^ The focus should be on training healthcare professionals throughout the nutrition care process according to the available resources and identified priorities. This requires achieving a balance between “best known” standards of care and the “best available” standards. It may be possible to establish partnerships between major clinical nutrition scientific societies from high‐income countries and national societies in LMICs to adapt existing training programs on nutrition care; translation to local languages may be required.

### Strengthening health systems

In RLSs, decision‐makers include local health authorities, international donors, and NGOs. These decision‐makers will need to understand that high‐quality, equitable nutrition care implementation is necessary to tackle DRM.

Nutrition care implementation should include basic hospital resources, a reliable supply chain for essential nutrition therapy and equipment, and a plan for human resource development. Access to nutrition care should be considered as a right linked to the human rights to food and to health.^26^

## RECOMMENDATIONS

**Figure 1:**
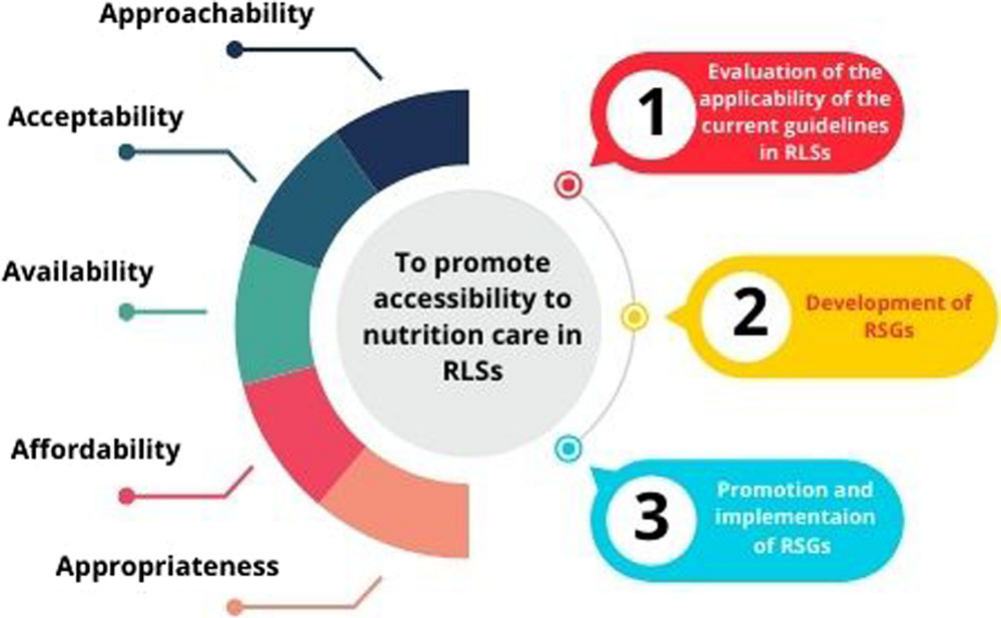
The three‐step strategy to address the identified barriers and promote access to nutrition care in RLSs. RLS, resources‐limited setting; RSG, resource‐stratified guideline.

The WG developed this position paper to address the challenges of disease‐related nutrition care in RLSs. The WG recognizes that addressing DRM in RLSs is challenging and will require sustained action. These actions need to be founded on the human rights–based approach while maintaining a patient‐centered approach to care. Both approaches identify the malnourished patient as a vulnerable person and highlight the responsibility of all stakeholders. Thus, the WG, on behalf of the clinical nutrition societies, recommends a three‐step strategy for tackling DRM and promoting nutrition care access in RLSs (Figure 1).

1. *Evaluation of the applicability of the current guidelines*. The experts agreed on the importance of evaluating the applicability of the current guidelines in RLS as a preliminary step. For this purpose, an online survey will be sent to the practitioners in the field of clinical nutrition in RLS that previously responded to the initial survey. The survey will evaluate the applicability of ESPEN guidelines to RLSs, providing valuable insight into the particular needs and barriers in RLSs.
2. *Development of RSGs*. The WG will lead the development of a RSG using the four resource levels: basic, limited, enhanced, and maximal resource settings.
3. *Promotion and implementation of RSGs*. The WG and the clinical nutrition societies will promote the RSG in clinical nutrition and will support its implementation in RLSs.

## CONCLUSIONS

Tackling DRM in RLSs is challenging because of the high burden of malnutrition and the fact that current guidelines may not be fully applicable in DRM. The WG recommends a three‐step strategy to promote access to nutrition care, including the development of RSGs.

## AUTHOR CONTRIBUTIONS

Diana Cardenas contributed to the conception and methodology of the data. Ines Ribeiro Ferreira, Maria Isabel Toulson Davisson Correia, Filomena Gomes, Mario Barbagallo, and Rocco Barazzoni equally contributed to the design of the survey; all the authors contributed to the acquisition and analysis of the data; Diana Cardenas drafted the manuscript; and all authors provided critical revision of the manuscript. Diana Cardenas, Ines Ribeiro Ferreira, Maria Isabel Toulson Davisson Correia, Filomena Gomes, Rocco Barazzoni, Simon Lal, Mario Barbagallo, and the RLS Working Group participated in the consensus meeting and critical revision of the manuscript. All authors critically revised the manuscript, agree to be fully accountable for ensuring the integrity and accuracy of the work, and read and approved the final manuscript.

## CONFLICT OF INTEREST STATEMENT

None declared.
